# Open peer review, pros and cons from the perspective of an early career researcher

**DOI:** 10.1128/mbio.01948-23

**Published:** 2023-10-09

**Authors:** Tania Henriquez

**Affiliations:** 1 Department of Medical Biotechnologies, University of Siena, Siena, Italy; Georgia Institute of Technology, Atlanta, Georgia, USA

## Abstract

Peer review is considered by many to be a fundamental component of scientific publishing. In this context, open peer review (OPR) has gained popularity in recent years as a tool to increase transparency, rigor, and inclusivity in research. But how does OPR really affect the review process? How does OPR impact specific groups, such as early career researchers? This editorial explores and discusses these aspects as well as some suggested actions for journals.

## EDITORIAL

Peer review, or the evaluation of submissions to an academic journal by a panel of reviewers in the same subject area, is considered by many to be a fundamental component in scientific publishing. This process was formally introduced by the Royal Society of Edinburgh in 1731 (1), although different sources trace its origins to 1665 (Henry Oldenburg) (2). Since its inception, peer review has evolved, adapting to times and disciplines. In this context, open peer review (OPR) has gained popularity in recent years as a tool to increase transparency, rigor, and inclusivity in research. But what is OPR? As described by Ross-Hellauer in 2017 (3), OPR is an umbrella term encompassing several peer review models consistent with Open Science (Table 1). These models can include one or more of the following terms: (i) open identities, (ii) open reports, (iii) open participation, (iv) open interaction, and (v) open pre-review manuscripts; being “open identities” and “open reports” the main traits of OPR (4). In this line, Ross-Hellauer reported that there are at least 22 different combinations or definitions for OPR in the literature (3). In this context, one of the most interesting definitions (currently used by the journal *eLife*) is a consultative review, in which reviewers and editors “consult with one another before sending out a decision after peer review” (5).

**TABLE 1:** Characteristics of main OPR models

OPR type	Characteristics
Open identities	Authors and reviewers know their identities, and this could happen from the beginning or after the acceptance of the manuscript
Open reports	Reports are published alongside manuscripts
Open participation	Wider scientific community can contribute to review
Open interaction	Direct communication between authors, reviewers, and editors
Open pre-review manuscript	Manuscript available before peer review

While OPR has gained traction in recent years in the biological sciences, it is not known how its implementation impacts the review process and how some groups, such as early career researchers, may be particularly affected (positively or negatively). Certainly, perspectives on OPR vary depending on whether the early career researcher is an author or a reviewer. For the early career author, OPR could be beneficial by increasing reviewer accountability, which can improve the overall quality of reports and make comments more constructive (6). In addition, the possibility of publishing supplementary material, such as response letters, could also allow authors to provide additional information without modifying or disturbing the flow of ideas in the main manuscript. However, it could also be a concern for young investigators who are new to publishing and might not want to share their initial response letters. Also, OPRs that are overwhelmingly negative or, in some cases, rude and demeaning may impact the confidence of early career authors as they can be broadly viewed online. In this case, consultative review may be a good model since discussion between editors and reviewers to generate a single report would, in theory, avoid the possibility of a biased or coarse report. Nevertheless, the editor and associated personnel should define criteria for which the OPR is constructive or not.

On the other hand, in the case of early career reviewers, OPR provides tangible evidence of the time and effort invested in the report (since some studies indicate that a reviewer spends an average of 8.5 hours on a single review (median of 5 hours) (7)). This recognition is especially important for early career researchers to advance in their careers (4). In this sense, it has been proposed that assigning DOIs to review reports, as mentioned by CrossRef (https://www.crossref.org/documentation/research-nexus/peer-reviews/) (4), can function as proof of the reviewers' contribution in perpetuity. However, it is not clear whether this will become a common practice. Another advantage in the case of open reports (which can contain some or all the review reports, responses and discussions, cover letters, etc.) is that they can be used as educational materials, providing valuable resources regarding the publication process and how to conduct a review or write a response letter. This is especially useful for younger generations, as not all early career researchers have the opportunity to receive formal training on this topic (although new initiatives, such as the mBio Junior Editorial Board, are establishing successful frameworks to tackle these inequities).

Early career researchers do not necessarily benefit from signing their reports (open identities). The system, unfortunately, is not perfect, and concerns have been raised regarding the negative impact that a signed review could have on junior researchers who are “dependent on senior scientists for opportunities and advancements” (https://plos.org/resource/open-peer-review /). Also, it is still not known how an open system would impact the reception of review reports made by women or underrepresented groups in science. In this context, it seems relevant that individual reviewers can decide for themselves whether to sign a review or not based on their own evaluation of the pros and cons of OPR (Table 2).

**TABLE 2:** Some pros and cons of OPR as early career researchers (ECR)

	Pros	Cons
**Author ECR**	Fair and transparent evaluation of one’s own work	Publication of response letters as inexperienced author Some reviewers are rude or not scientifically sounding. Publishing inappropriate bad comments may negatively influence the future career of the young researcher
**Reviewer ECR**	Recognition of review reports Access to additional educational materials	Negative impact of reviews performed on seniors’ works (retaliation?) Negative reception of reviews made by certain groups (discrimination?)

Perhaps journals should allow authors and reviewers to make their choice between all these different combinations of OPR and see, over time, what kind of preference each community has and how each choice impacts the different actors in the process. In this context, it is important that journals develop forms of data collection and monitoring that allow a rigorous evaluation of the information provided by the scientific community.

Will OPR solve all the problems associated with peer review? Probably not, but it seems worthwhile to test which option might be best for each scenario and how we can progressively improve the system to make it more transparent and fairer for all.
